# Assessment of Oral Health Conditions Among Physically Disabled Patients in Saudi Arabia

**DOI:** 10.1016/j.identj.2024.06.020

**Published:** 2024-07-23

**Authors:** Abdulaziz A. Alharbi, Adel M. Al Shehri, Fawaz H. Alzahrani, Hammad F. Turkstani, Bandar S. Shukr, Mohammed A. Alzubaidi, Mohammed F. Felemban

**Affiliations:** a Department of Oral and Maxillofacial Surgery and Diagnostic Sciences, Faculty of Dentistry, Taif University, Taif, Saudi Arabia; b Armed Forces Center for Health Rehabilitation, Armed Forces Hospitals in Taif Region, Taif, Saudi Arabia; c Faculty of Dentistry, Taif University, Taif, Saudi Arabia; d Department of Preventive Dentistry, Faculty of Dentistry, Taif University, Taif, Saudi Arabia

**Keywords:** DMFT, CPITN, OHI-S, Disabled individuals, Oral health

## Abstract

**Objectives:**

This study was undertaken to assess the oral health status among physically disabled Saudi patients.

**Methods:**

Recruitment took place in the Armed Forces Centre for Health Rehabilitation in Taif, Saudi Arabia. 124 patients living with a physical disability were enrolled and divided into three groups: hemiplegia, paraplegia and quadriplegia. Data was collected on demographics and different oral indices, including the Decayed Missing Filled Teeth (DMFT) index, the Mean Decayed Teeth score, the Community Periodontal Index (CPI), the Community Periodontal Index of Treatment Needs (CPITN), and the Simplified Oral Hygiene Index (OHI-S).

**Results:**

High DMFT was observed among the hemiplegia group (mean = 22.61; *P*-value = .008) with no difference in the Mean Decayed Teeth. All groups showed signs of gingivitis in the form of gingival bleeding. The most compromised periodontal health in the form of pockets 6 mm or deeper was found in the hemiplegia group (53.7%; *P*-value = .001). This was also reflected in the CPITN (39.0%; *P*-value = .001), indicating the need for complex treatments in the same group. Regarding oral hygiene, all groups showed a “fair” oral hygiene condition (OHI-S < 3.0), with significantly poorer hygiene (mean = 2.49; *P*-value = .042) and greater debris accumulation (mean = 1.52; *P*-value = .024) among the quadriplegia group. In the regression analysis, both age and gender had significant effects on some indices, while disability type showed borderline effects.

**Conclusion:**

The findings indicate poor oral health in these individuals, mainly due to physical limitations, hindering effective self-care practices.

**Clinical significance:**

Oral health is a critical aspect in people with physical disabilities, as it is intrinsically linked to overall health and well-being. Recognizing the clinical importance of oral health among physically disabled patients is essential to improve access and affordability of dental care for this vulnerable group of the population.

## Introduction

According to the World Health Organization (WHO), a disability can be defined as an outcome the interaction of three elements: a health condition, either physical (e.g., cerebral palsy) or mental (e.g., depression, Down syndrome), personal factors (e.g., negative attitudes) and environmental factors (e.g., lack of social support, limited transportation).^1^ According to the WHO, worldwide, one billion people have some form of disability, accounting for approximately 15% of the global population.^2^ Saudi Arabia is a developing country in the Middle Eastern region. In 2016, approximately 667,280 Saudi nationals reported having a disability, resulting in a prevalence rate of 3326 per 100,000 people (3.3%).^3^

Recent reports have shown the presence of health disparities between people with disabilities and the general population.^4^^,^^5^ Oral health is an important aspect, and studies have highlighted poorer oral health among individuals with disabilities compared with their healthy counterparts.^6^^,^^7^ Common indicators for poor dental health in people with disabilities include a high prevalence of untreated dental caries, periodontal disease, missing teeth, prolonged retention of deciduous teeth, supernumerary teeth, and malocclusions.^8^ The consequences of poor oral health are enormous. Recent studies have demonstrated that poor dental health can affect overall health, with significant correlations to major chronic conditions such as diabetes, cardiovascular disease, and respiratory disease.^9^^,^^10^ Moreover, the quality of life of individuals is considerably impacted by their oral health, particularly psychological and social aspects.^11^^,^^12^ For instance, poor dental health can result in decreased nutrient intake owing to problems with chewing and digestion, tooth pain, anxiety, difficulties in completing daily tasks, impacted facial appearance and speech, and poor social relations.^11^, ^12^, ^13^

Compared with those without impairments, people who have disabilities are more susceptible to poor oral health and tend to have complex oral health demands.^14^ This increased demand is mainly attributed to multiple risk factors, such as limited access to health care services, the need for aid to perform basic oral hygiene tasks (e.g., brushing the teeth), difficulties in communication, and a greater possibility of having lower levels of both income and education in comparison with healthy individuals.^15^, ^16^, ^17^ Additionally, the oral health condition could be worse in people with limited oral hygiene practices as a result of motor, sensory, or intellectual disabilities.^18^

In recent years, several studies have been published on the dental health of the general public of Saudi Arabia.^19^^,^^20^ However, the dental health of physically and intellectually challenged people has received little attention, despite the fact that they require specialized care. With almost a billion individuals living with some form of disability around the world according to the WHO statistics, scientific studies on disabilities are critical to help international health initiatives understand the unique oral health difficulties experienced by this vulnerable sub-population, such as health inequality, limited access to healthcare, and subsequent poor quality of life. Additionally, these studies contribute to raising awareness among healthcare providers and policymakers in an effort to prompt actions from local and international health organizations and develop inclusive and accessible healthcare strategies and interventions. In an attempt to meet some of these goals and contribute to the body of literature, particularly in the country of Saudi Arabia, this study was undertaken to examine the oral health status and the prevalence of common dental conditions among a sample of Saudi individuals living with physical disabilities. In addition, this study aimed to compare the dental health status of various physical disability groups (hemiplegia, paraplegia, and quadriplegia).

## Materials and methods

### Study design and ethical approval

This descriptive cross-sectional study evaluated the oral health condition of a group of Saudi and non-Saudi patients with disabilities. The data were collected from February to May 2023. Before enrolment in the study, an informed consent form, written in the Arabic language, was obtained from all participants who were capable of understanding. In addition, written consent was obtained from parents/legal guardians for those who had limitations in comprehending the information in the form. This study received approval from the institution's Research Ethics Committee (Approval No. H-02-T-078). Furthermore, this manuscript was prepared according to the STROBE (Strengthening the Reporting of Observational Studies in Epidemiology) criteria.^21^

### Study sample

The study was conducted in the Armed Forces Centre for Health Rehabilitation in Taif, Saudi Arabia. This centre is one of the main comprehensive rehabilitations centres in the city and is a part of the Armed Forces Hospital. The centre houses a maximum of 139 patients of both sexes who are ≥20 years of age, all with either a physical or mental disability or both. Furthermore, the centre is equipped with a small dental clinic to perform simple dental procedures, and patients who need complex treatments are referred to the dental department at the Armed Forces Hospital.

Cooperative patients with physical disabilities who were diagnosed with hemiplegia, paraplegia, or quadriplegia were included in the study. *“Hemiplegia”* or “*hemiparesis”* is a unilateral paralysis that affects only one side of the body.^22^ The term *“paraplegia”* refers to the lack of sensation or movement in the legs and lower trunk.^23^ *“Quadriplegia,”* on the contrary, implies the partial or total paralysis of the torso and all four extremities.^24^ To minimize potential risks and difficulties related to the oral examination, patients with a recent episode of epilepsy, those fed via a nasogastric tube, and those who were potentially uncooperative were excluded. The inclusion and exclusion criteria were framed after consulting the centre's medical team and reviewing the current literature.^25^ Those who had recently experienced an epileptic seizure were excluded because any stress could trigger another seizure during the oral examination.^25^ In addition, performing an examination is difficult in patients using a nasogastric tube, those who are potentially uncooperative, such as patients with a self-harming tendency, and those suffering from severe learning disability.^25^ The study participants were voluntarily selected using a convenient sampling method. At the time of data collection, a total of 139 patients were staying at the rehabilitation centre. The study aimed to include all the patients in the facility. Using the "Raoasft" website, sample size estimations were calculated with a 5% margin of error and a 95% confidence interval, and it was estimated to be minimally 103 participants. Because some patients met the exclusion criteria (e.g., a recent episode of epilepsy) (*n* = 15), the study sample comprised 124 participants categorized into three groups: 1- hemiplegia (*n* = 41); 2-paraplegia (*n* = 43); 3-quadriplegia (*n* = 40).

### Data collection

To ensure consistent understanding, documentation, and interpretation of the survey criteria and codes, all examiners were trained before collecting the research data by a team of experts from two institutions. Two examiners volunteered to participate in the data collection process and were named *“Examiner A”* and *“Examiner B*.” Both examiners participated in a training course that lasted for 1 week using photos and radiographs from patient volunteers.

Demographic and clinical data were collected using the standardized *World Health Organization Oral Health Survey*.^26^ The survey was modified according to the study objectives. Demographic information included age, sex, type of disability and its cause. Clinical information comprised caries and periodontal diagnoses as well as plaque and calculus examinations. Before the clinical examinations, the participants’ teeth were neither brushed nor professionally cleaned. The prevalence of caries was assessed using the *Decayed Missing Filled Teeth (DMFT)* index and the mean score of decayed teeth.^26^^,^^27^ The conservative concept of *“score sound when in doubt”* was applied during dental caries examination, and the tooth was classified as “carious” only if enamel destruction or cavitation was evident.

Periodontal status was evaluated using the modified *Community Periodontal Index (CPI)*, which includes two main indicators: gingival bleeding and periodontal pockets.^26^ All teeth present in the mouth were examined and probed using the WHO CPI periodontal probe. In addition, the *Community Periodontal Index of Treatment Needs (CPITN)* was recorded to classify the different disability groups according to their treatment requirements.^26^^,^^28^ For this index, the dentition was categorized into six parts (sextants), and each one was given a code number. The indexed teeth in each sextant were examined, and only the worst score was recorded. Moreover, the sextant was included in the examination only if two or more functioning teeth were present and not indicated for extraction.

Clinical examination also included the recording of the *Simplified Oral Hygiene Index (OHI-S)* as an indicator of oral cleanliness, which comprised the measurement of both *Simplified Debris Index (DI-S)* and *Simplified Calculus Index (CI-S)*.^26^^,^^29^ The overall OHI-S score was calculated by adding the scores of DI-S and CI-S. Subsequently, the OHI-S score was interpreted as good (0.0-1.2), fair (1.3-3.0) or poor (3.1-6.0). For patients diagnosed with hemiplegia or paraplegia, all clinical examinations were performed at the dental chair using the unit's light and dry air sources, an examination kit (mirror, tweezer and explorer), and a WHO CPI periodontal probe. Nonetheless, patients with quadriplegia could not be examined on the dental chair; therefore, they were seen at the bedside using a portable light source, a chip blower for dry air, a disposable examination kit, and a WHO CPI periodontal probe. Each examiner was accompanied by a recorder who documented all the necessary information. For each clinical examination, universal infection control measures were adopted and rigorously followed.

### Statistical analysis

Sample characteristics were presented as means and standard deviations or as frequencies and percentages, as appropriate. The chi-square test and one-way ANOVA were used to compare the different groups with disabilities in terms of various dental indexes. Alternatively, in some comparisons, the Fisher's exact test was applied instead of the chi-square test in case of nonparametric data. In ANOVA, both Kolmogorov–Smirnov test and the Q–Q plot were used to evaluate the normality of the outcomes, whereas Levene's test was used to assess the equality of variances. Furthermore, multiple comparison using Tukey's test was performed only if ANOVA was significant in identifying which groups differed significantly in the analysed outcome. No violations were detected in the data regarding the normality or equality of variances when ANOVA was performed.

In addition, adjusted linear regression models were used to evaluate the associations between the type of disability and the DMFT index, mean decayed teeth score, as well as OHI-S, while adjusted logistic regression models were used in the assessment of both CPI and CPITN scores. Findings were presented as adjusted beta coefficients for linear models, and adjusted odds ratios (AORs) for logistic models, along with the corresponding 95% confidence intervals (CIs). All models were adjusted for age and sex, and the Variance Inflation Factor (VIF) was utilized to identify any multi-collinearity issues between the variables. Additionally, influential outliers were identified using Cook's distance. The Hosmer and Lemeshow test were used to assess the models’ fitness in the logistic regression, while the adjusted Coefficient of Determination (Adj. R2) was used in the linear regression. No violations were identified regarding predictors’ collinearity, influential observations or model fitness. However, some models (i.e., CPI for gingival bleeding, CPITN) produced incorrect estimates due to the presence of an insufficient number of participants in some categories, therefore, collapsing was performed when feasible.^30^ All collected data were analysed using the Statistical Package for Social Sciences (SPSS) software (Version 25.0, Chicago, IL, USA). All statistical tests were two-sided, and statistical significance was set at a *P*-value of ≤ .05.

## Results

**Fig. 1:**
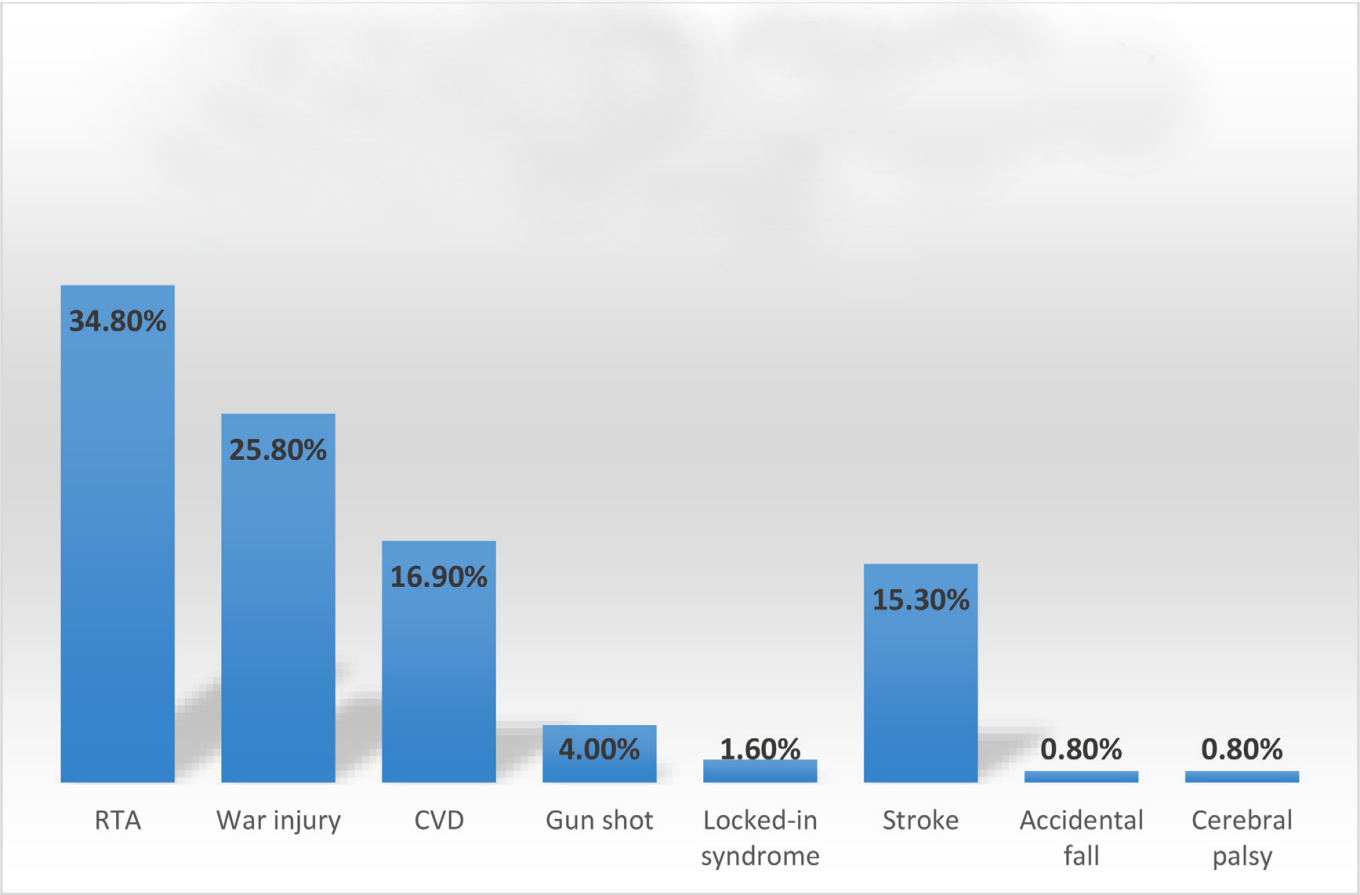
Causes of physical disability in the study sample.

Overall, the study sample comprised 124 participants (109 men and 15 women) in the age group of 20-88 years. The participants were categorized into three groups according to the type of disability in their medical records, with approximately the same percentage of participants in each group (hemiplegia: 33%; paraplegia: 34.7%; quadriplegia: 32.3%). In addition, Figure 1 highlights the different causes of physical disability reported by the study participants, with the highest percentage (34.80%) of disabilities attributed to road traffic accident (RTA), followed by war injury (25.80%), and the lowest percentage (0.80%) due to cerebral palsy.

Table 1 illustrates the mean DMFT and the mean decayed teeth scores in each group according to the disability type. The overall DMFT was 20.15, with a significant mean difference among the three groups (*P*-value = .008). The highest DMFT mean score was found in the hemiplegia group (DMFT = 22.61). Findings from the multiple comparison Tukey's test indicated that subjects with hemiplegia had a significantly higher mean DMFT score than those with paraplegia (mean difference = 5.19, *P*-value = .006). The overall mean decayed teeth score was 12.96, with the highest score found in those diagnosed with quadriplegia (mean decayed teeth = 14.35). However, no significant differences were observed among the different disability groups.

**Table 1:** Mean DMFT scores and Mean Decayed Score according to the type of disability.

Diagnosis		DMFT	Mean Decayed Score
Hemiplegia (*n* = 41)	Mean	22.61	12.39
	Std. Deviation	7.36	6.45
Paraplegia (*n* = 43)	Mean	17.42	12.21
	Std. Deviation	8.42	7.43
Quadriplegia (*n* = 40)	Mean	20.55	14.35
	Std. Deviation	6.86	7.68
Total (*N)*	Mean	20.15	12.96
	Std. Deviation	7.84	7.21
	*P*-value (ANOVA)	.008*	.335

⁎ *P-*value ≤ .05 was considered statistically significant.

Regarding modified CPI, most participants (98.4%) had a CPI score of 1 (bleeding) in gingival bleeding and a similar score (pocket 4-5 mm) in periodontal pockets (53.2%). These results indicate the prevalence of both gingival and periodontal diseases among the study participants. No significant differences were observed between the disability groups in the CPI scores for gingival bleeding. Nevertheless, the groups differed significantly in the CPI scores for periodontal pockets (*P*-value = .001). Specifically, periodontal pockets of 4-5 mm were more prevalent in subjects with paraplegia (60.5%) and quadriplegia (62.5%) than in those with hemiplegia (36.6%). On the contrary, deeper pockets (≥6 mm) were more common in subjects diagnosed with hemiplegia (53.7%) than in those diagnosed with paraplegia (11.6%) or quadriplegia (12.5%) (Table 2). Regarding CPITN, most study participants (78.2%) had a score of TN-2, which signified the need for professional scaling, removal of plaque retentive factors, and oral hygiene instructions. When examining the different disability groups, however, significant differences were noted (*P*-value = .001). A score of TN-2 was more common in subjects with paraplegia (83.7%) and quadriplegia (92.5%) than in those with hemiplegia (58.5%). Conversely, a score of TN-3, which indicates the need for complex treatment involving deep scaling and root planning, was more common in those with hemiplegia (39.0%) than in those with paraplegia (9.3%) or quadriplegia (7.5%) (Table 3).

**Table 2:** The modified Community Periodontal Index (CPI) scores according to the type of disability.

Diagnosis	CPI scores for gingival bleeding			
	CPI 0 (healthy) *n* (%)	CPI 1 (bleeding) *n* (%)	CPI X (tooth excluded/not present) *n* (%)	
Hemiplegia (*n* = 41)	2 (4.9)	39 (95.1)	-	
Paraplegia (*n* = 43)	0 (0.0)	43 (100.0)	-	
Quadriplegia (*n* = 40)	0 (0.0)	40 (100.0)	-	
Total (*N*)	2 (1.6)	122 (98.4)	-	
**Fisher's exact**	**value**		***P***-**value (2-sided)**	
	2.72		.210	
	CPI scores for periodontal pockets			
Diagnosis	CPI 0 (healthy) *n* (%)	CPI 1 (pocket 4-5 mm) *n* (%)	CPI 2 (pocket 6 mm or more) *n* (%)	CPI X (tooth excluded/not present) *n* (%)

Hemiplegia (*n* = 41)	4 (9.8)	15 (36.6)	22 (53.7)	-
Paraplegia (*n* = 43)	12 (27.9)	26 (60.5)	5 (11.6)	-
Quadriplegia (*n* = 40)	10 (25.0)	25 (62.5)	5 (12.5)	-
Total (*N*)	26 (21.0)	66 (53.2)	32 (25.8)	-
**Pearson Chi-square**	**value**		***P*-value (2-sided)**	
	25.37		.001*	

⁎ *P*-value ≤ .05 was considered statistically significant.

**Table 3:** The Community Periodontal Index of Treatment Needs (CPITN) classification based on the type of disability.

Diagnosis	CPITN category			
	TN-0 (no treatment)	TN-1 (improvement of personal oral hygiene)	TN-2 (professional scaling and removal of plaque retentive factors and oral hygiene instructions)	TN-3 (complex treatment which can involve deep scaling-root planning, complex procedures)
Hemiplegia (*n* = 41)	1 (2.4)	0 (0.0)	24 (58.5)	16 (39.0)
Paraplegia (*n* = 43)	0 (0.0)	3 (7.0)	36 (83.7)	4 (9.3)
Quadriplegia (*n* = 40)	0 (0.0)	0 (0.0)	37 (92.5)	3 (7.5)
Total (*N*)	1 (0.8)	3 (2.4)	97 (78.2)	23 (18.5)
**Fisher's exact**	**value**		*P*-value (2-sided)*	
	21.03		.001*	

⁎ *P*-value ≤ .05 was considered statistically significant.

In most examined participants, the oral hygiene status, as assessed using OHI-S, was found to be “fair” (OHI-S = 2.23). There was a significant difference (*P*-value = .024) between the groups in terms of DI-S. Multiple comparison Tukey's test revealed significantly higher debris scores in subjects with quadriplegia than in those with hemiplegia (mean difference = 0.36, *P*-value = .031). In addition, those with paraplegia exhibited higher debris scores than those with hemiplegia; however, the finding was only borderline significant (mean difference = 0.30, *P*-value = .078). Higher calculus scores were also noted in subjects with quadriplegia (CI-S = 0.96) than in those with hemiplegia (CI-S = 0.71) or paraplegia (CI-S = 0.86); nonetheless, this finding was not statistically significant. Moreover, the disability groups differed significantly in OHI-S (*P*-value = .042). The Tukey's test showed higher OHI-S scores in subjects diagnosed with quadriplegia than in those with hemiplegia (mean difference = 0.61, *P*-value = .041) (Table 4).

**Table 4:** Mean DI-S, CI-S and OHI-S scores according to the type of disability.

Diagnosis		DI-S	CI-S	OHI-S
Hemiplegia (*n* = 41)	Mean	1.16	0.71	1.88
	Std. Deviation	0.65	0.49	1.09
Paraplegia (*n* = 43)	Mean	1.46	0.86	2.33
	Std. Deviation	0.68	0.59	1.17
Quadriplegia (*n* = 40)	Mean	1.52	0.96	2.49
	Std. Deviation	0.58	0.59	1.09
Total (*N*)	Mean	1.38	0.84	2.23
	Std. Deviation	0.65	0.56	1.14
	*P*-value (ANOVA)	.024*	.141	.042*

⁎ *P*-value ≤ .05 was considered statistically significant.

Findings from the adjusted regression analyses revealed a borderline significant effect (*P*-value = .084) for the type of disability on the average DMFT index. Specifically, those with quadriplegia had an average DMFT score that was 2.56 points higher than those with paraplegia. In addition, age had a significant effect on the DMFT index (*P*-value = .001). The results showed that for every one-unit (year) increase in age, the average DMFT score increased by 0.23. The type of disability did not have any significant influences on the mean decayed teeth score; however, sex showed a significant effect (*P*-value = .012), with female participants having an average decayed teeth score that was 5.01 lower than males. Additionally, age had a borderline significant effect on decayed teeth score (*P*-value = .060), with an increase in the mean score by 0.07 for every one-unit (year) increase in age. No significant effects were observed for the type of disability or other predictors on the average OHI-S score (Table 5).

**Table 5:** Multivariable linear regression analysis evaluating the association between the type of disability and the different oral health indices (DMFT index, Mean Decayed Score, OHI-S).

Parameter	DMFT index		Mean Decayed Score		OHI-S	
	β coefficient (95% CI)	*P*-value	β coefficient (95% CI)	*P*-value	β coefficient (95% CI)	*P*-value
Age	0.23 (0.16 to 0.31)	.001*	0.07 (-.03 to 0.15)	.060	0.01 (-0.01 to 0.01)	.882
Sex (male)	Reference	Reference	Reference	Reference	Reference	Reference
Sex (female)	-2.02 (-5.74 to 1.70)	.285	-5.01 (-8.93 to -1.10)	.012*	-0.39 (-1.02 to 0.23)	.218
Disability type (hemiplegia)	0.75 (-2.56 to 4.07)	.652	-0.50 (-3.98 to 2.98)	.775	-0.40 (-0.96 to 0.15)	.159
Disability type (paraplegia)	Reference	Reference	Reference	Reference	Reference	Reference
Disability type (quadriplegia)	2.56 (-.34 to 5.47)	.084	2.19 (-0.86 to 5.24)	.159	0.18 (-0.31 to 0.67)	.469

⁎ *P*-value ≤ .05 was considered statistically significant.

The type of disability, as well as sex, did not have any significant impacts on the outcome of CPI for both gingival bleeding and periodontal pockets. However, age had a borderline significant effect (*P*-value = .064) on the CPI for periodontal pockets (4-5 mm), with every one-unit increase in age, the odds of having a pocket 4-5 mm are expected to increase by 1.03. Likewise, age had a significant effect (*P*-value = .004) on the CPI for periodontal pockets (6 mm or more), with an increase in the odds of having a pocket 6 mm in depth or more by 1.07 for every one-unit increase in age. The findings showed a borderline significant effect (*P*-value = 0.074) for the type of disability on the CPITN. Namely, the odds of having a TN-3 (complex periodontal treatment needs) score in the CPITN were 3.35 more in patients with hemiplegia compared to those with paraplegia. In addition, age was significantly associated (*P*-value = .036) with CPITN, with 1.03 higher odds of having a TN-3 score for every one-unit increase in age (Table 6).

**Table 6:** Multivariable logistic regression analysis evaluating the association between the type of disability and the different oral health indices (CPI, CPITN).

Parameter	CPI (gingival bleeding)◆		CPI (periodontal pockets)¶		CPI (periodontal pockets)ɸ		CPITN¥	
	AOR (95% CI)	*P*-value	AOR (95% CI)	*P*-value	AOR (95% CI)	*P*-value	AOR (95% CI)	*P*-value
Age	0.85 (0.70 to 1.02)	.093	1.03 (0.99 to 1.07)	.064	1.07 (1.02 to 1.13)	.004 *	1.03 (1.00 to 1.06)	.036 *
Sex (male)	Reference	Reference	Reference	Reference	Reference	Reference	Reference	Reference
Sex (female)	7.80E + 07 (0.00 to ∞)	.998	1.37 (0.24 to 7.58)	.751	1.17 (0.10 to 13.36)	.897	1.38 (0.35 to 5.45)	.638
Disability type (hemiplegia)	0.00 (0.00 to ∞)	.997	1.01 (0.24 to 4.26)	.981	3.12 (0.50 to 19.22)	.220	3.35 (0.89 to 12.63)	.074
Disability type (paraplegia)	Reference	Reference	Reference	Reference	Reference	Reference	Reference	Reference
Disability type (quadriplegia)	0.20 (0.00 to ∞)	1.00	0.98 (0.34 to 2.79)	.983	1.04 (0.19 to 5.55)	.959	0.74 (0.15 to 3.66)	.781

AOR = adjusted odds ratio.

⁎ *P*-value ≤ .05 was considered statistically significant.

◆ Dependent variable: CPI scores for gingival bleeding: CPI 1 (bleeding) vs CPI 0 (healthy).

¶ Dependent variable: CPI scores for periodontal pockets: CPI 1 (pocket 4-5 mm) vs CPI 0 (healthy).

ɸ Dependent variable: CPI scores for periodontal pockets: CPI 2 (pocket 6 mm or more) vs CPI 0 (healthy).

¥ Dependent variable: CPITN category: TN-3 vs TN-0/TN-1/TN-2 (few-number categories were collapsed).

## Discussion

Achieving and maintaining optimum oral health in people with disabilities is a critical aspect of their adequate overall wellbeing. However, this remains an area with significant obstacles and inequalities. Few studies assessing the oral health condition of individuals with disabilities in Saudi Arabia are available in the literature.^25^^,^^31^^,^^32^ The current research is unique as it evaluates and compares the oral health conditions of a sample of individuals having physical disabilities and diagnosed with hemiplegia, paraplegia, or quadriplegia in Saudi Arabia. The findings revealed a significantly high DMFT score among participants with hemiplegia and a high experience of caries (mean decayed teeth score) in those with quadriplegia. Regarding gingival and periodontal health, all three groups showed signs of gingivitis, mainly gingival bleeding. The worst periodontal condition was deep pockets observed in individuals with hemiplegia. This finding was also reflected in CPITN, and the need for complex treatment procedures was the highest in this group (hemiplegia). In terms of the oral hygiene status, all three groups displayed a “fair” oral hygiene condition, with significantly poorer hygiene and greater accumulation of debris in individuals with quadriplegia.

Physical disability is a disorder that greatly affects a person's mobility, physical capacity, and/or motor functions, hindering their capacity to perform daily tasks. Such disabilities are caused by either congenital/hereditary or acquired factors.^33^^,^^34^ Physical disabilities that occur later in life fall within the category of acquired disabilities. According to the WHO, RTA is one of the leading causes of acquired physical disabilities, accounting for 20-50 million nonfatal injuries globally.^35^ The findings of this study are consistent with the current literature as most participants reported RTA to be the reason for their disability.

The overall oral health of people with physical disability can vary owing to several factors, including the extent of disability and the individual's capability to perform personal hygiene tasks such as oral care. In this study, three main types of physical disability, in the form of paralysis affecting various areas of the body, were investigated: hemiplegia, paraplegia, and quadriplegia. As expected, the findings showed that among these disability groups, individuals with paraplegia had fewer dental issues and better overall oral health. This observation could be explained by the fact that their disability affected only the lower limbs. Unlike hemiplegia or quadriplegia, this disability does not directly affect arm function. Therefore, the patients can use their hands to perform routine oral care practices, such as brushing, flossing, and mouth washing, independently.^36^ Conversely, compromised oral health conditions in the form of high caries experience, as denoted by DMFT, and frequent accumulations of both calculus and dental plaque, as indicated by both DI-S and CI-S, were observed among patients with quadriplegia. A possible reason is that this type of disability significantly limits the individual's ability to perform oral hygiene independently because of the paralysis involving all four limbs. Therefore, the patients often need assistance from others, such as caregivers.^36^^,^^37^ In addition, studies have shown that reliance on others for performing routine oral hygiene tasks exacerbates the risk of developing oral health issues.^36^ Moreover, a recent investigation by Moldvai et al. suggested a possible link between Functional Independence Measure (FIM) and oral health conditions. Individuals with lower FIM scores (indicating high dependence) might brush their teeth less often, leading to poorer oral health.^37^

Hemiplegia is a form of paralysis that affects half of the body and involves an arm and a leg.^38^ Although the extent of paralysis varies, losing function in one arm could make it difficult for the affected person to perform essential oral hygiene tasks. This difficulty could exert a negative impact on the oral health condition.^36^ In this study, participants with hemiplegia had the most compromised periodontal status in the form of pockets ≥6 mm in depth (*P*-value = .001). The poor periodontal status was also indicated by CPITN, and there was a significantly higher proportion of individuals in the TN-3 category. This finding emphasizes the need for more complex treatment compared with the other disability groups (*P*-value = .001). One possible explanation for this observation is that these individuals experience limited mobility and dexterity, which poses challenges in brushing their teeth or using the dental floss in coordinated movements or in reaching all areas of their mouth. In addition, they might experience a reduction in the normal salivary secretion, particularly in the paralyzed half of the mouth, or because of the use of medications that induce xerostomia or dry mouth, especially in patients with stroke. Other explanations include negligent behaviour toward oral health as a result of dependency on others, depression, or lack of motivation. Although people with paraplegia may have better overall dental health than those with hemiplegia or quadriplegia, every person's condition is different and various factors can affect an individual's oral health status.

Findings from the present study showed that older age was a significant predictor for higher DMFT score, deeper periodontal pockets (6 mm or more), as well as complex periodontal treatment needs. One possible explanation is the interplay of multiple factors in elderly people, including the cumulative exposure to different risk factors over time (e.g., inadequate care practices, unhealthy dietary habits, smoking), reduction in the saliva either due to the physiological aging process or medication use, and periodontal diseases and gingival recessions caused by the aging process or chronic illness (e.g., diabetes).^39^ Moreover, older individuals typically have more dental restorations, some of which can be complex, and they might become defective over time.^39^ Together, all these factors can contribute to the development of dental caries and periodontal disease, leading to the need for complex periodontal interventions. The results also showed that female participants had significantly lower mean decayed teeth compared to males. This was consistent with a recent study of non-disabled elderly population in Poland.^40^ This phenomenon can be explained by the potential differences in various biological and lifestyle factors. Gender differences in saliva composition might exist, possibly influencing the person's ability to balance the acidic environment and reverse early carious lesions. In addition, compared to women, men might prefer to consume a diet that is acidic and rich in sugar, which can increase their risk of developing dental caries and erosions. Individual habits and access to dental care also play significant roles. Women typically practice better oral hygiene compared to men. This was evident in a large nation-wide study that enrolled 17,632 subjects from 56 countries and reported more adherence to oral hygiene standards among women compared to men.^41^ Moreover, the research supports the fact that women may possess better oral health literacy in comparison to men, therefore, they are more proactive about seeking preventive dental treatment.^42^

This study has several strengths. This research is unique as it investigates the oral health status of a neglected subpopulation in Saudi Arabia, namely, people with physical disabilities. A comprehensive data collection method that involved both clinical examination and standardized survey administration was adopted in an attempt to provide a comprehensive view of the oral health status and the challenges faced by such individuals. Furthermore, this work was intended to raise awareness among dental professionals, caregivers, disability experts, and policymakers about the importance of oral health in this vulnerable subpopulation. Additionally, the study sample included respondents with different types of physical disabilities and was relatively adequate in size (*N* = 124). Nevertheless, this study also has certain limitations. First, it was assumed that the oral health and treatment needs of the excluded subjects (e.g., uncooperative patients and those with recent epilepsy) would not be better than those of the included subjects. However, such an assumption may not be completely accurate. Second, both CPI and CPITN measures can only evaluate health outcomes that respond to treatment.^43^ These are not quite useful when assessing irreversible or untreatable outcomes, such as gingival recession and attachment level, and they cannot be used as markers of disease activity.^43^ Third, as DMFT considers both filled and missing teeth, it is a useful tool for estimating past caries experiences but not present treatment requirements. In addition, estimates produced by indexes (such as CPITN and OHI-S) that utilize indexed teeth may be either overestimated or underestimated and do not accurately represent the state of oral health. Fourth, the study findings would be more useful if the participants were compared regarding oral hygiene practices to see if this has any effect on the present oral health condition (e.g., participants who had adequate oral hygiene habits might have better oral health compared to those who had poor or unsatisfactory habits), in addition to exploring the effect of different demographic factors through a multivariable analysis. Lastly, this study was a single-centre observational study. Therefore, multicentre studies with larger samples are needed in the future to precisely understand the oral health condition and the influencing factors in this vulnerable group of the Saudi population as well as in similar populations.

## Conclusion

In summary, physical disability in the form of hemiplegia, paraplegia, or quadriplegia can lead to compromised oral health because of mobility limitations, difficulty in performing oral care tasks, and increased dependency on others. Overall, the oral health status of the subjects with disabilities examined in this study was relatively poor. Dental caries and periodontal disease were highly prevalent, heavy calculus and plaque accumulations were noted, and the treatment needs were complex in the different disability groups.

## Conflict of interest

The authors declare that they have no known competing financial interests or personal relationships that could have appeared to influence the work reported in this paper.
